# A Community‐Engaged Approach to Recruit the Brazilian Population in the US: Initial Lessons from the BRAINY‐NJ Study (Brazilian Aging in New York ‐ New Jersey Study)

**DOI:** 10.1002/alz70860_100602

**Published:** 2025-12-23

**Authors:** Ana Staniscia, Karen Cai, Rebecca West, Yaakov Stern, Monica G Rivera Mindt, Sonia Maria Dozzi Brucki, Michal S Beeri, Sharon Sanz Simon

**Affiliations:** ^1^ New Jersey Medical School, Rutgers University, Newark, NJ, USA; ^2^ Herbert and Jacqueline Krieger Klein Alzheimer's Research Center at Rutgers Brain Health Institute, New Brunswick, NJ, USA; ^3^ Herbert and Jacqueline Krieger Klein Alzheimer's Research Center at Rutgers Brain Health Institute, New Brunswick, NJ, USA; ^4^ Columbia University Vagelos Collège of Physicians and Surgeons, New York, NY, USA; ^5^ Icahn School of Medicine, Mount Sinai Hospital, New York, NY, USA; ^6^ Fordham University, New York, NY, USA; ^7^ University of São Paulo School of Medicine, São Paulo, São Paulo, Brazil

## Abstract

**Background:**

The Brazilian population in the US is growing and aging but remains underrepresented in US health research. In addition to group‐specific genetic and environmental risks, the Brazilian immigrant population in the US likely presents risks for health inequities due to limited healthcare access/utilization and immigration‐related stress. Language has been a barrier to recruiting the Brazilian‐US population as Portuguese‐speaking populations are not typically included in the US‐Latinx cohorts.

**Method:**

The BRAINY‐NJ cohort study is being initiated to investigate the impact of cardiovascular and lifestyle/sociocultural risk factors on cognition and brain function in 120 cognitively healthy Brazilian immigrants (55+) living in/around the New York City area (New Jersey / New York). The study follows a community‐based participatory research (CBPR) paradigm to formalize the academic‐community partnership through different actions: 1) establishing partnerships with the community organizations and leaderships through in‐person events; 2) building a research team culturally sensitive and proficient in Portuguese; 3) collecting data at the events to assess community perceptions, interests, and priorities; 4) identify potential members for the study's Community Advisory Board; 5) identify community resources to recruit participants.

**Result:**

Over 10 months, the BRAINY‐NJ organized three in‐person events: one health fair and two educational 2.5‐hour meetings. The events were in Portuguese and on weekends. The educational meetings included: 1) Educational component on brain/mental health, dementia prevention, and the BRAINY‐NJ; 2) Social component(e.g., breakfast, Brazilian live music, and dance); and 3) Assessment of interests and suggestions. The educational meetings included 30‐40 participants, the main topics of interest were aging, memory, sleep, and emotional health. Most participants (87%) reported never having participated in a study, and 93.3% reported interest in joining the study. More participants rated cognitive/emotional health as a main concern rather than physical health. The BRAINY‐NJ study partners with local NGOs, and Brazilian faith‐based organizations (e.g., Churches and Afro‐Brazilian Umbanda Center).

**Conclusion:**

Recruitment of underrepresented populations, including the Brazilian‐US population, is urgently needed. In‐person events are critical to engage a community with limited research in the US. Research activities that connect topics on brain/cognitive health with emotional health seem particularly of interest to the Brazilian immigrant community.

